# Spatial compartmentalization at the nuclear periphery characterized by genome-wide mapping

**DOI:** 10.1186/1756-8935-6-S1-P126

**Published:** 2013-04-08

**Authors:** Feinan Wu, Jie Yao

**Affiliations:** 1 Department of Cell Biology, Yale University School of Medicine, 333 Cedar Street, New Haven, CT 06520, USA

## Background

How gene positioning to the nuclear periphery regulates transcription remains largely unclear. By high-resolution cell imaging, we have previously observed the differential compartmentalization of transcription factors and histone modifications at the nuclear periphery in mouse C2C12 myoblasts [1]. Here, we aim to identify DNA sequences associated with the nuclear lamina (NL) and examine this compartmentalization at the genome-wide level.

## Materials and methods

We cultured NIH-3T3 mouse fibroblasts and C2C12 mouse myoblasts, performed DNA adenine methyltransferase identification (DamID) assay on mouse Lamin B1 as previously described [2], and sequenced the methylated DNA fragments using high-throughput DNA sequencing at Yale Center for Genome Analysis. We have developed a set of bioinformatic analyses to identify and compare genomic regions and/or genes with enriched adenine methylation.

## Results

We have identified ~15, 000 sequencing-based Lamina-Associated Domains (sLADs) in both mouse 3T3 fibroblasts and C2C12 myoblasts. These genomic regions range from a few kb to over 1 Mb and cover ~30% of the genome. We have used immunofluorescence staining and DNA-FISH to verify that all of the tested regions are spatially proximal to the NL. We have analyzed the sLAD regions relative to genome-wide distributions of histone modifications in C2C12 myoblasts [3-5]. We found that genomic regions covered by sLADs are characterized by extremely low levels of active histone modifications such as H3K4me2/3, H3K9Ac, H3K36me3 and H3K79me2, but sLAD and non-sLAD regions contain similar densities of repressed histone modifications such as H3K27me3.

We have also cross-analyzed the available gene expression data in C2C12 myoblasts [6] with the NL-association map generated in this study, and identified 272 expressed genes in significant association with the NL that we named as active sLAD genes. Genomic regions around transcription start sites of active sLAD genes display reduced associations with the NL and possess active histone modifications. Surprisingly, we noticed that gene bodies of active sLAD genes possess very low levels of active histone modifications such as H3K36me3.

## Conclusions

Our experimental results and bioinformatic analyses in mouse C2C12 myoblasts suggest that chromatin at the nuclear periphery is characterized by the paucity of active histone modifications rather than the enrichment of repressive histone modifications. The distinct histone modification profiles among active sLAD genes may give clues on how gene positioning to the nuclear periphery (via NL association) affects transcription regulation in mammalian cells.
